# Factors Associated with Quality of Life (QoL) in Adolescents and Young Adults with Cancer

**DOI:** 10.3390/curroncol32090475

**Published:** 2025-08-22

**Authors:** Reanna George, Geoff Eaton, Pam Crotty, Cheryl Heykoop, Norma D’Agostino, Sapna Oberoi, Joshua A. Rash, Sheila N. Garland

**Affiliations:** 1 Department of Psychology, Faculty of Science, Memorial University, St. John’s, NL A1B 3X9, Canada; regeorge@mun.ca (R.G.); jarash@mun.ca (J.A.R.); 2 Beatrice Hunter Cancer Research Institute, Halifax, NS B3H 0A2, Canada; 3 Young Adult Cancer Canada (YACC), St. John’s, NL A1A5B5, Canada; geoff@youngadultcancer.ca (G.E.); pam@youngadultcancer.ca (P.C.); 4 Faculty of Leadership Studies, Royal Roads University, Victoria, BC V9B 5Y2, Canada; cheryl.1heykoop@royalroads.ca; 5 Department of Supportive Care, Princess Margaret Cancer Centre, University Health Network, Toronto, ON M5G 2M9, Canada; 6 Department of Pediatrics and Child Health, University of Manitoba, Winnipeg, MB R3A 1S1, Canada; soberoi@cancercare.mb.ca; 7 Department of Pediatric Hematology-Oncology, CancerCare Manitoba, Winnipeg, MB R3E 0V9, Canada; 8 Discipline of Oncology, Faculty of Medicine, Memorial University, St. John’s, NL A1B 3X5, Canada

**Keywords:** quality of life (QoL), adolescents and young adults (AYAs), oncology

## Abstract

Adolescents and young adults (AYAs) diagnosed with cancer between the ages of 15 and 39 years face unique challenges that impact their long-term quality of life (QoL). Physical functioning, relationship status, social support, anxiety, and depression have been associated with QoL in AYAs with cancer. This study explored factors associated with increased QoL in a sample of 392 AYAs with cancer in Canada who participated in the initial Young Adult Cancer Canada RECOVER survey. The EORTC QLQ-C30 was used to measure QoL. Adjusting for relevant demographic, cancer, and clinical variables, the only factors significantly associated with QoL in multivariable analysis were symptoms of depression [Mild (*β* = −0.420, *p* < 0.001); Moderate (*β* = −0.937, *p* < 0.001); Moderately Severe (*β* = −1.188, *p* < 0.001); Severe (*β* = −2.182, *p* < 0.001)] and generalized anxiety [Mild (*β* = −0.244, *p* = 0.012); Moderate (*β* = −0.420, *p* = 0.002); Moderately severe (*β* = −0.400, *p* = 0.012); Severe (*β* = −0.697, *p* = 0.010)], as well as having completed treatment (*β* = −0.347, *p* < 0.001). Age, gender, time since diagnosis, having children, education, income, fear of recurrence, and social support were not significantly associated with QoL. These results support the need for age-appropriate resources to help AYAs manage the long-term psychological impacts of cancer.

## Simple Summary

Young adults between ages 15–39 who are diagnosed with cancer face distinct challenges that can significantly impact their long-term well-being. While previous research has identified various factors like physical health, relationships, and social support as important for quality of life in this population, this study sought to clarify which factors matter most. Among 392 young adults with cancer in Canada, mental health symptoms were the strongest predictors of quality of life—the more severe the symptoms, the worse the quality of life. Age, gender, income, social support, and fear of cancer returning were not significantly associated with quality-of-life once psychological factors were considered. The results should inform the prioritization of age-appropriate psychological resources, potentially improving outcomes during and after cancer treatment.

## 1. Introduction

A cancer diagnosis in adolescence and young adulthood (between 15 and 39 years of age) can interrupt important stages of development and compromise identity formation, education and professional aspirations, relationships and family planning, and financial independence [1,2,3]. Although there has been progress, adolescents and young adults (AYAs) remain an underrepresented population in research [4]. Overall survival rates for cancer are increasing, but not for those in the AYA age group where many cancers have a worse prognosis [4,5]. This recognition has led to calls from advocacy and research organizations to investigate the immediate and long-term impacts of cancer on this population.

Quality of life (QoL) as an umbrella concept encompasses many aspects of physical, emotional, social, and role functioning [6]. There is initial evidence that the long-term QoL of AYAs is compromised. Compared to an age and sex matched comparison group, only 68.2% of a sample of 195 AYA cancer post treatment reported good physical health and 49.2% reported good mental health compared to 90.6% and 89.8% of their peers, respectively [7]. When compared to a sample of 1509 older cancer survivors, AYAs (n = 81) report greater impairment in QoL than adults who were 50 years of age and older [8]. Further, 46% of this sample of AYAs reported clinical impairment in QoL at the beginning of the study, 3 months, 6 months, and 12 months follow up. Given that the majority of AYAs diagnosed with cancer will ideally live longer as cancer survivors than they did prior to their diagnosis, QoL is a critical measure of recovery [9].

Poorer QoL in AYAs has been associated with higher levels of fatigue, poor sleep, pain, and psychological distress during treatment and into survivorship [10]. Psychological factors that have been associated with reduced QoL include fear of cancer recurrence, cognitive concerns, anxiety, and depression [7,11]. Given the developmentally vulnerable life stage of AYAs, social and functional concerns also have a large impact on QoL, such as being single, poor body image, and future family planning [7,12]. Social support appears to have a nuanced impact on QoL with some aspects of support (e.g., peer supports, and family support including support from older children) being associated with better QoL among those who experienced colorectal, lung and breast cancer [6,13,14]. These types of support were often associated with physical and emotional aspects of QoL. However, there is research suggesting that higher perceived social support in a Canadian AYA population has a more negative relationship to physical functioning [7].

Understanding QoL is necessary so that resources can be allocated accordingly [15]. This study aims to describe the QoL of a heterogeneous sample of Canadian AYAs and explore demographic and clinical factors associated with QoL.

## 2. Materials and Methods

### 2.1. Participants

This study uses data from the initial web-based survey from YACC’s RECOVER study, which examines the long-term impacts of cancer in the lives of young adults. The study was approved by the Community Research Ethics Office (CREO-314) and Memorial University’s Health Research Ethics Board (20241398). The Young Adult Cancer Canada (YACC) Recover Study is a community led, mixed-methods longitudinal study that seeks to engage AYAs with lived/living experience with cancer to understand QoL over time (5-year follow-up). Participants were eligible to participate in RECOVER if they were 18 years of age and older, had received a cancer diagnosis before the age of 40 years, were living in Canada, and could understand English or French. Data used in these analyses was collected between 21 September 2023, and 27 January 2025. Data collection and follow-up are still ongoing.

Participants were recruited through the following methods: (1) printed advertisements and provider referral; (2) mail-out cancer registry invitations in the provinces of Alberta and Manitoba; (3) mail outs to potentially eligible participants in the Atlantic Partnership for Tomorrow’s Health project; (4) social media such as X and Facebook (5) media releases on local radio stations, print and television programs; and (6) though the YACC community. Informed consent was obtained electronically by all participants via the Qualtrics platform. The survey included measures that looked at QoL, which is the focus of the current study, but also measures of resilience, depression, anxiety, financial impact, fear of recurrence, and social provisions. The survey took approximately 30–45 min to complete. The survey platform allowed participants to skip any question they wished not to answer. Each participant was offered a CAD 20 digital gift card for participating.

### 2.2. Measures

The EORTC QLQ-C30 questionnaire was used to obtain information on participant’s QoL. The EORTC QLQ-C30 consists of 30 questions which comprises five functional subscales (physical, role, emotional, social, and cognitive functioning) and eight symptom scales (fatigue, pain, and emesis; a global health status subscale; and six single items to assess the financial impact and symptoms such as dyspnea, sleep disturbance, appetite, diarrhea, and constipation). Items on each scale are summed and transformed onto a scale of 0–100. Higher scores on the global health status or functional subscales represent higher QoL, while higher scores on symptom scales (e.g., fatigue) represent higher levels of problems and lower QoL. The summary score was also calculated based on recommendations and validation in prior research [16,17]. Each global and functional subscale score and the summary score were categorized by normal (76–100), mild (51–75), moderate (26–50), and severe (0–25) impact on QoL. Each symptom subscale was categorized as normal (0–25), mild (26–50), moderate (51–75), and severe (76–100) impact on QoL based on scoring suggested by Saini et al. [18]. The summary score was used as the dependent variable for the current analysis.

Patient Health Questionnaire (PHQ-9), a 9-item self-report tool to measure symptoms associated with depression based in the DSM-IV criteria [19]. The Generalized Anxiety Disorder (GAD-7) is a self-report measure used to assess the severity of symptoms associated with generalized anxiety disorder [20]. The Fear of Cancer Recurrence Inventory—Short Form (FCRI-SF) includes 9-items to measure severity of fear of cancer recurrence. The Social Provisions Scale (SPS-5), a 5-item self-report measure, was used to examine perceived level of social support. Finally, questions on household (“What was your total family (household) income in CAD before taxes in the last year (include wages, salaries and self-employment earnings)?”) and patient (“What percentage of family income was earned by you (the patient) last year?”) earned income were taken from the Patient Self-Administered Financial Effects (P-Safe) measure [21].

### 2.3. Analysis

There was no missing data for the EORTC QLQ-C30 (the dependent variable). Exploration of the remaining measures (PHQ-9, GAD-7, DCRI-SF, and SPS-5) revealed that data was not missing at random, Little’s MCAR = χ^2^(1195) = 1524.58, *p* < 0.001. Exploration of variables indicated greater proportions of missing data among variables that were presented near the end of the survey, suggesting that the pattern of missingness was most likely due to response fatigue. The variable with the most missing values was P-Safe at 11.1% missing with <5% of missing values evidenced across other independent variables (i.e., PHQ-9, GAD-7, FCRI-SF, and SPS-5). Missing values were handled using single imputation with the Expectation-Maximization (EM) algorithm in the Missing Values Analysis package for SPSS version 26.

Descriptive statistics were used to describe the demographic, cancer related, and clinical characteristics of the sample. To determine factors associated with lower QoL, variables (i.e., demographic, cancer related, and clinical characteristics) were entered into univariable linear regression models. To investigate the unique association between such predictors and QoL, covariates yielding association with *p* values < 0.05 were entered into a multivariable linear regression model. Collinearity was assessed for the predictors in the multivariable regression model, using Variance Inflation Factors (VIF) and tolerance, all of which were within the accepted range, indicating that multicollinearity was not a concern for this model. All regression models were conducted with Jamovi version 2.2.12.

To ensure valid, representative, and that responses were provided by humans, we implemented a rigorous process which included a pre-screening questionnaire. Narrative response questions were embedded within the survey to distinguish genuine participants from automated responses or computer-generated bots. The main survey also included Qualtrics’ security features, including CAPTCHA, IP address monitoring, response time tracking, and attention checks to help identify and exclude bot-generated or fraudulent responses, as well as a manual review of the flagged responses and data cleaning to ensure the dataset was accurate, reliable, and aligned with the study’s target demographic.

## 3. Results

Overall, 676 participants consented to participate in the RECOVER study; however, for this analysis, we restricted participants to those who were diagnosed with cancer between the ages of 15–39 years and who were between the ages of 18–39 years at the time of survey completion. Furthermore, those who completed less than 50% of the survey were also excluded (n = 12). This resulted in a current sample of 392. The average age of the sample was 32.69 (SD = 4.78) years, with the majority of the sample identifying as women (71.4%) and who described their racial background as white (70.7%). Over half of the sample had an undergraduate degree (55.0%) and a small portion of the sample reported currently attending school (16.1%).

Within the dataset, the most common type of cancer was hematologic/blood cancers (29.3%) followed by head and neck (20.9%), breast (20.2%), and gynecological (9.4%) cancer. The mean age at the time of their cancer diagnosis was 28.6 years and 68.5% reported finishing their cancer treatment. Only 10.1% reported having had a recurrence of cancer. Of participants, 45.4% reported their cancer having a negative impact on their QoL. Complete demographic information can be found in Table 1.

The global QoL subscale score was 66.62 (SD = 18.37), indicating mild impairment in overall QoL. The sample reported a moderate level of financial impairment (Mean 30.19, SD = 39.94), and mild impairment in their emotional, cognitive, social, and role functioning. Respondents scored in the normal range for physical functioning. On the symptom scales, mild QoL impairment was caused by fatigue, pain, insomnia, and constipation. Scores relating to nausea and vomiting, dyspnea, appetite loss, and diarrhea were in the normal range. The EORTC QLQ-C30 subscales are summarized in Table 2.

Separate univariable linear regressions were used to identify significant independent factors associated with EORTC QLQ C-30 summed scores. At the univariable level, several demographic factors were associated with higher QoL, including having children (*β* = 0.474, *p* < 0.001), being 5–9 years since first diagnosis (*β* = 0.665, *p* = 0.020), having completed treatment (*β* = 0.570, *p* < 0.001). Having an undergraduate degree (*β* = −0.465, *p* = 0.014) was associated with having a lower QoL. Proportion of household income earned was also associated with QoL. Respondents who earned 25–49% of the household income (*β* = 0.442, *p* = 0.002), and 50–74% of the household income (*β* = 0.353, *p* = 0.010) reporting higher QoL. A dose–response relationship was observed whereby more severe symptoms of depression were associated with increasingly lower quality-of-life scores [Mild (*β* = −0.641, *p* < 0.001); Moderate (*β* = −1.304, *p* < 0.001); Moderately Severe (*β* = −1.622, *p* < 0.001); Severe (*β* = −2.837, *p* < 0.001)]. A dose–response relationship was observed whereby more severe symptoms of anxiety were associated with increasingly lower quality-of-life scores [Mild (*β* = −0.652, *p* < 0.001); Moderate (*β* = −1.125, *p* < 0.001); Moderately Severe (*β* = −1.582, *p* < 0.001); Severe (*β* = −2.166, *p* < 0.001)]. Clinically severe fear of cancer recurrence (FCRI-SF) was significantly associated with lower QoL (*β* = −0.687, *p* = 0.045). Social support (SPS-5) was also significantly associated with higher QoL at the univariable level (*β* = 0.154, *p* = 0.002).

The multivariable linear regression included all of the variables that were significant in the univariable models. The overall model was significant (*F* = 18.76, *p* < 0.001) with an adjusted R^2^ of 0.544, and a RMSE value of 11.66. Review of the residual plots suggested that the data fits a linear model. In the multivariable model, severity of symptoms of depression [Mild (*β* = −0.420, *p* < 0.001); Moderate (*β* = −0.937, *p* < 0.001); Moderately Severe (*β* = −1.188, *p* < 0.001); Severe (*β* = −2.182, *p* < 0.001)] and severity of symptoms of anxiety [Mild (*β* = −0.244, *p* = 0.012); Moderate (*β* = −0.420, *p* = 0.002); Moderately severe (*β* = −0.400, *p* = 0.012); Severe (*β* = −0.697, *p* = 0.010)] were associated with worse QoL. Having completed treatment (*β* = −0.347, *p* < 0.001) was significantly associated with better QoL. Adding time since diagnosis and currently completed treatment as a separate interaction term to the multivariable model did not appreciably change the results. A summary of the regression models can be found in Table 3.

## 4. Discussion

The current study sought to understand QoL among AYAs in Canada and investigate the factors associated with QoL. This is important because QoL serves as a general assessment of well-being, capturing multiple facets of functioning. Although the mean EORTC QLQ-C30 score places the sample in the mild impairment range, close to half of the participants in this study had some level of impairment in QoL. This is consistent with the observation made by Schulte et al. that AYAs had lower QoL than an age and sex matched non-cancer comparison sample [7]. The psychological burden of this sample was high, with 41% endorsing clinical levels of FCR, 30% reporting moderate to severe levels of depression, and 27% reporting moderate to severe anxiety. In a previous study of 461 AYA with cancer in Canada, 59% of participants reported scores on the FCRI-SF > 22, indicating clinical levels of FCR, which was associated with psychological distress [22]. A systematic review of 34 studies of FCR in AYA reported that levels of clinical FCR ranged from 21 to 93% and was significantly associated with other psychological concerns including anxiety [23]. Almost 50% of this sample was more than 5 years past their diagnosis emphasizing that the psychological impacts of cancer last well into survivorship. We can no longer avoid addressing the long-term psychological impact of cancer and urgently need to ensure that the evidence-based interventions to address these concerns and improve QoL are scaled for implementation in clinical practice.

Our results suggest that financial QoL is among the areas most negatively impacted among AYAs diagnosed with cancer. The mean scores of the current sample are below the EORTC QLQ-C30 sensitivity scores suggested by Lidington et al. to identify young adults with cancer who would benefit from financial aid and other supports [24]. The Young Adults with Cancer in their Prime (YACPRIME) study also highlighted the disruptive financial impact of cancer, reporting that AYA survivors were more likely to have outstanding debt and less likely to own assets when compared to age, sex, and education matched peers [25]. There was also a positive univariate relationship between the percentage of patient-earned income and QoL, though this association was no longer significant after accounting for symptoms of depression and anxiety. Patient income can have implications in the type of care that they receive. In an international online survey of 283 cancer survivors, one of the most impactful barriers to obtaining psychosocial care was reported to be the financial costs [26]. The financial impact of cancer is important to discuss as this is a Canadian sample where all participants benefit from a government funded universal health care system, yet participants are still financially impacted by a cancer diagnosis. Additionally, AYAs need interventions that decreases the financial impact of cancer. However, for them to be beneficial to AYAs these intervention need to be tested in the target population for their effectiveness to ensure resources that are put in place effectively meet the unique needs of the AYA population [27]. More research is needed to understand the full scope of the financial impacts of cancer on long-term AYA survivors.

The strongest associations observed in multivariable models were between QoL and severity of symptoms of depression and anxiety. There is a clear dose–response relationship between these psychological concerns and QoL wherein as severity of symptoms of anxiety and depression increase, there was a pronounced decrease in QoL. Mystakidou et al. observed a similar inverse relationship in a sample of 120 participants with various cancer diagnoses (ages of 18–83 years) between various subscales of the EORTC QLQ -C30, including global QoL, and anxiety and depression scores on the Hospital Anxiety and Depression Scale (HADS) [11]. This association also emerged following interviews of 23 AYAs, caregivers and health care providers [28]. Among the common themes related to QoL, psychosocial well-being was identified by AYAs as an important component of QoL. Anxiety is not only a predictor of reduced QoL in AYAs with breast cancer, but also their relatives [29]. Psychological distress is a significant barrier to recovery in AYAs, and appropriate psychological supports are required during and after cancer treatment to help improve QoL among AYAs [15,26,30]. Our results also suggest that completing treatment is more strongly associated with QoL than time since diagnosis. Symptom management may play an important role in patients QoL during treatment, [31] but additional research is needed to understand these interactions.

This study has several strengths including a relatively large and heterogeneous sample, the use of robust and well-validated measurement tools, and representation from across Canada. Another strength of this study is that data had a low percentage of missing data, and imputation allowed for the use of as many responses as possible. The primary disadvantage of this study was the limited representativeness of underrepresented respondents. Those with higher levels of distress may be more likely to participate in research that investigates QoL in order to express their concerns. This limitation can only be overcome if research is conducted nationally through a coordinated system of cooperating cancer centers and follow-up is maintained past discharge. Unfortunately, such resource allocation does not yet exist in Canada, making cross-sectional surveys necessary. Comparing AYAs diagnosed with cancer to an age- and gender-matched comparison group could provide further insight and should be prioritized for future research. Further, the majority of the sample (71.4%) identified as women. Despite efforts to enroll more men and gender-diverse people, more work is necessary to engage and build trust with groups that are under-represented in research. This might be performed through recruitment in partnership with gender-inclusive organizations, and by sharing results that are specific to those populations. Response tendency and participants’ answering patterns were also not measured, which could have impacted the results. Although the use of single imputation allowed for a complete dataset, it can underestimate variability and introduce bias. This may have been a particular concern for the financial measures as it had the largest amount of missing data. Future research should investigate additional sociodemographic factors related to QoL and the impacts of different social supports on QoL for this age group.

## 5. Conclusions

QoL is impaired in close to 50% of AYAs with cancer, long after they complete treatment. The largest contributors to a reduced QoL in AYAs were levels of psychological distress. Age-appropriate supports may include community- or hospital-based in-person or virtual peer support groups, individual- or group-based psychotherapeutic interventions, or digital health interventions that address the issues that are most significant for AYAs. Having a variety of evidence-based options is important to accommodate diverse needs and personal treatment preferences. Having accessible and age-appropriate supports in place for managing anxiety and depression related to a cancer diagnosis in AYAs would help improve QoL and overall recovery in this understudied population.

## Figures and Tables

**Table 1:** Demographic and clinical descriptives.

Variable (*N* = 392)		N (%) or Mean (SD)
Age	Range: 20–39 years	Mean = 32.69 (SD: 4.78)
	20–29 years	102 (26.0%)
	29–34 years	120 (30.6%)
	34–39 years	170 (43.4%)
Gender	Gender Diverse	17 (4.3%)
	Men	95 (24.2%)
	Women	280 (71.4%)
Racial Background	Pacific Islander	1 (0.3%)
	Hispanic	2 (0.5%)
	Other	7 (1.8%)
	Indigenous	9 (2.3%)
	Black	16 (4.1%)
	Multiracial	17 (4.3%)
	Asian	63 (16.1%)
	White	277 (70.7%)
Region	Territories (YT, NT, NU)	1 (0.3%)
	Atlantic (PEI, NS, NB, NL)	24 (6.1%)
	Western (BC)	49 (12.5%)
	Central (ON, QC)	122 (31.1%)
	Prairies (AB, SK, MB)	196 (50.0%)
Have Children	Yes	133 (34.1%)
	No	257 (65.9%)
In a Relationship	No	119 (30.6%)
	Yes	270 (69.4%)
Highest education	High school or less	53 (13.9%)
	Some post-secondary	57 (14.9%)
	Graduate degree or more	62 (16.2%)
	Undergraduate degree	210 (55.0%)
Currently in school	Yes	63 (16.1%)
	No	329 (83.9%)
Worked in the past 12 months	Yes	336 (85.7%)
	No	53 (14.3%)
Household income (P-Safe)	Less than CAD 19,000	17 (5.2%)
	Less than CAD 49,000	38 (11.7%)
	Less than CAD 99,000	145 (44.8%)
	More than CAD 100,000	124 (38.3%)
Patient earned income (P-Safe)	0–24%	89 (22.7%)
	25–49%	105 (26.8%)
	50–74%	120 (30.6%)
	75–100%	78 (19.9%)
Have a family doctor	No	24 (6.1%)
	Yes	368 (93.9%)
Cancer Type *	Skin	12 (3.1%)
	Gastrointestinal	19 (4.8%)
	Bone and soft tissue	19 (4.8%)
	Genitourinary	29 (7.4%)
	Gynecological	37 (9.4%)
	Breast	79 (20.2%)
	Head and neck	82 (20.9%)
	Hematologic/Blood	115 (29.3%)
Age at diagnosis (years)		Mean = 27.64 (SD = 5.51)
	15–24 years	110 (28.1%)
	25–29 years	114 (29.1%)
	30–34 years	131 (33.4%)
	35–40 years	37 (9.4%)
Time Since Diagnosis		Mean = 4.74 (SD = 2.79)
	<2 years	13 (3.3%)
	2–4 years	189 (48.2%)
	5–9 years	151 (38.5%)
	10+ years	39 (9.9%)
Treatment	Haven’t received treatment	21 (5.4%)
	Currently undergoing treatment	102 (26.1%)
	Completed treatment	268 (68.5%)
Completed treatment	Within the last 2 years	125 (47.5%)
	Within the last 5 years	87 (33.1%)
	Within the last 10+ years	51 (19.4%)
Cancer recurrence	Yes	39 (10.1%)
	No	347 (89.9%)
QoL		Mean = 73.69 (SD = 18.09)
	Normal (76–100)	214 (54.6%)
	Mild (51–75)	141 (36.0%)
	Moderate (26–50)	29 (7.4%)
	Severe (0–25)	8 (2.0%)
Depression		Mean = 7.99 (SD = 5.82)
	None	128 (32.7%)
	Mild	145 (37.0%)
	Moderate	61 (15.6%)
	Moderately Severe	39 (9.9%)
	Severe	19 (4.8%)
Anxiety		Mean = 7.33 (SD = 5.39)
	None	155 (39.5%)
	Mild	130 (33.2%)
	Moderate	53 (13.5%)
	Moderately Severe	45 (11.1%)
	Severe	9 (2.3%)
Fear of Recurrence		Mean = 19.92 (SD = 6.73)
	Non-clinical	45 (11.5%)
	Possibly clinical	57 (14.5%)
	Likely clinical	131 (33.4%)
	Clinical severity	159 (40.6%)
Social Support		17.02 (2.76)

* Gastrointestinal includes colon/colorectal and rectal cancers. Gynecological includes kidney and testicular cancers. Head and neck includes thyroid, brain/CNS, lung and salivatory cancers. Hematologic/blood includes non-Hodgkin lymphomas, Hodgkin lymphomas, and leukemia. Skin includes melanomas. Bone and soft tissue include sarcomas.

**Table 2:** EORTC QLQ-C30 subscales summary.

Subscales		Mean (SD)	Level of Impairment
Global QoL		66.62 (18.37)	Mild
Financial Difficulties		30.19 (34.94)	Moderate
Functional Scales	Physical functioning	85.94 (17.40)	Normal
	Role functioning	69.90 (29.09)	Mild
	Emotional functioning	59.82 (25.31)	Mild
	Cognitive functioning	71.43 (27.36)	Mild
	Social functioning	66.96 (28.54)	Mild
Symptom Scales	Fatigue	40.22 (27.75)	Mild
	Nausea and vomiting	10.50 (18.91)	Normal
	Pain	25.43 (27.72)	Mild
	Dyspnea	15.99 (24.42)	Normal
	Insomnia	42.18 (33.05)	Mild
	Appetite loss	23.72 (28.73)	Normal
	Constipation	26.04 (18.91)	Mild
	Diarrhea	11.99 (23.86)	Normal

Each global and functional subscale score and the summary score were categorized by normal (76–100), mild (51–75), moderate (26–50), and severe (0–25) impact on QoL. Each symptom subscale was categorized by normal (0–25), mild (26–50), moderate (51–75), and severe (76–100) impact on QoL based on scoring suggested by Saini et al., (2024) [15].

**Table 3:** Linear regression of factors associated with quality of life.

		Univariable Linear Regression		Multivariable Linear Regression	
		*β* [95% CI]	*p*	*β* [95% CI]	*p*
Age	21–29 years	0			
	29–34 years	0.120 [−0.145, 0.385]	0.375		
	34–39 years	0.063 [−0.183, 0.310]	0.613		
Gender	Women	0			
	Men	0.102 [−0.130, 0.335]	0.388		
	Gender diverse?	−0.454 [−0.943, 0.036]	0.069		
Children	No	0			
	Yes	0.474 [0.269, 0.678]	**<0.001**	−0.113 [−0.050, 0.276]	0.174
Highest education	Highschool or less	0			
	Some post-secondary	−0.465 [−0.837, −0.094]	**0.014**	−0.009 [−0.291, 0.273]	0.950
	Undergraduate degree	−0.029 [−0.329, 0.271]	0.849	0.110 [−0.115, 0.366]	0.336
	Graduate degree or more	−0.212 [−0.577, 0.152]	0.253	0.076 [−0.202, 0.353]	0.593
Patient earned income	0–24%	0			
	25–49%	0.442 [0.164, 0.719]	**0.002**	0.046 [−0.171, 0.263]	0.676
	50–74%	0.353 [0.083, 0.622]	**0.010**	0.044 [−0.156, 0.245]	0.665
	75–100%	−0.081 [−0.380, 0.218]	0.593	−0.089 [−0.312, 0.134]	0.433
Time Since Diagnosis	<2 years	0			
	2–4 years	0.321 [−0.236, 0.878]	0.258	0.069 [−0.334, 0.472]	0.736
	5–9 years	0.665 [0.103, 1.226]	**0.020**	0.449 [−0.258, 0.583]	0.449
	10+ years	0.451 [−0.171, 1.073]	0.154	−0.023 [−0.478, 0.432]	0.920
Treatment	Currently on treatment	0			
	Haven’t received	0.123 [−0.334, 0.579]	0.598	0.193 [−0.165, 0.551]	0.289
	Completed treatment	0.570 [0.348, 0.791]	**<0.001**	0.347 [0.169, 0.525]	**<0.001**
Cancer recurrence	No	0			
	Yes	−0.384 [−0.715, −0.054]	**0.023**	−0.074 [−0.321, 0.174]	0.559
Depression	None	0			
	Mild	−0.641 [−0.476, −0.806]	**<0.001**	−0.420 [−0.226, −0.615]	**<0.001**
	Moderate	−1.304 [−1.516, −1.092]	**<0.001**	−0.937 [−1.197, −0.678]	**<0.001**
	Moderately Severe	−1.622 [−1.872, −1.373]	**<0.001**	−1.188 [−1.511, −0.865]	**<0.001**
	Severe	−2.837 [−3.172, −2.502]	**<0.001**	−2.182 [−2.628, −1.736]	**<0.001**
Anxiety	None	0			
	Mild	−0.652 [−0.842, −0.463]	**<0.001**	−0.244 [−0.433, −0.055]	**0.012**
	Moderate	−1.125 [−1.379, −0.871]	**<0.001**	−0.420 [−0.689, −0.152]	**0.002**
	Moderately severe	−1.582 [−1.852, −1.312]	**<0.001**	−0.400 [−0.710, −0.090]	**0.012**
	Severe	−2.166 [−2.713, −1.619]	**<0.001**	−0.697 [−1.225, −0.168]	**0.010**
Fear of cancer recurrence (FCRI-SF)	Non-clinical	0			
	Possibly clinical	0.107 [−0.269, 0.482]	0.577	−0.004 [−0.279, 0.271]	0.977
	Likely clinical	−0.314 [−0.640, −0.011]	0.058	−0.134 [−0.375, 0.106]	0.272
	Clinical severity	−0.687 [−1.004, −0.369]	**<0.001**	−0.060 [−0.305, 0.184]	0.628
Social support (SPS-5)		0.154 [0.056, 0.253]	**0.002**	0.016 [−0.060, 0.091]	0.686

All numbers in bold are significant at *p* < 0.05.

## Data Availability

After de-identification, individual participant data may be available following article publication to investigators whose proposed use of the data has been approved by an independent review committee for the purpose of individual participant data meta-analysis. To gain access, data requestors will need to sign a data access agreement.

## Abbreviations

The following abbreviations are used in this manuscript:

AYAs	Adolescents and Young adults
QoL	Quality of life
YACC	Young Adult Cancer Canada
